# Evaluation of Inflammatory Status in COVID-19 Patients with Chronic Kidney Disease: A Comparative Analysis Based on Creatinine Clearance Levels

**DOI:** 10.3390/biomedicines12122707

**Published:** 2024-11-27

**Authors:** Andreea Banta, Daniela Rosca, Ovidiu Rosca, Iulia Bogdan, Teodor Cerbulescu, Loredana Gabriela Stana, Elena Hogea, Daciana Nistor

**Affiliations:** 1 Doctoral School, “Victor Babes” University of Medicine and Pharmacy Timisoara, Eftimie Murgu Square 2, 300041 Timisoara, Romania; andreea.banta@umft.ro (A.B.); daniela.rosca@umft.ro (D.R.); 2 Discipline of Infectious Diseases, “Victor Babes” University of Medicine and Pharmacy Timisoara, Eftimie Murgu Square 2, 300041 Timisoara, Romania; ovidiu.rosca@umft.ro (O.R.); iulia-georgiana.bogdan@umft.ro (I.B.); 3 Department III—Microscopic Morphology, Discipline of Cellular and Molecular Biology, “Victor Babes” University of Medicine and Pharmacy, Eftimie Murgu Square 2, 300041 Timisoara, Romania; cerbulescuteodor28@gmail.com; 4 Department I, Discipline of Anatomy and Embriology, “Victor Babes” University of Medicine and Pharmacy, Eftimie Murgu Square 2, 300041 Timisoara, Romania; loredana.stana@umft.ro; 5 Discipline of Microbiology, “Victor Babes” University of Medicine and Pharmacy Timisoara, Eftimie Murgu Square 2, 300041 Timisoara, Romania; 6 Department of Functional Sciences, Physiology, Centre of Imuno-Physiology and Biotechnologies (CIFBIOTEH), “Victor Babes” University of Medicine and Pharmacy Timisoara, Eftimie Murgu Square 2, 300041 Timisoara, Romania; daciana_nistor@umft.ro

**Keywords:** COVID-19, chronic kidney disease, inflammatory status, creatinine clearance, inflammatory scores

## Abstract

Background and Objectives: Patients with chronic kidney disease (CKD) are at increased risk of severe COVID-19 outcomes due to their compromised immune systems and chronic inflammatory state. This study aimed to evaluate and compare the inflammatory status of COVID-19 patients with CKD, stratified by creatinine clearance (CrCl) levels: CrCl < 30 mL/min, CrCl 30–60 mL/min, and CrCl > 60 mL/min. Multiple inflammatory scores combining laboratory parameters were assessed, including novel scores and established indices. Methods: In this retrospective cohort study, 223 patients admitted with confirmed COVID-19 were included and divided into three groups based on CrCl levels: CrCl < 30 (n = 41), CrCl 30–60 (n = 78), and CrCl > 60 (n = 104). Laboratory parameters including C-reactive protein (CRP), interleukin-6 (IL-6), neutrophil-to-lymphocyte ratio (NLR), ferritin, platelet count, absolute neutrophil count (ANC), absolute lymphocyte count (ALC), and serum albumin were collected. Multiple inflammatory scores were calculated, including inflammation scores (IS1–IS4), the systemic inflammatory index (SII), the C-reactive protein-to-albumin ratio (CAR), the lymphocyte-to-C-reactive protein ratio (LCR), and the prognostic nutritional index (PNI). Statistical analyses were performed to compare inflammatory scores among groups and assess correlations with clinical outcomes. Results: The CrCl < 30 group exhibited significantly higher levels of inflammatory markers and inflammatory scores compared with the other groups (*p* < 0.001). Among the additional scores, CAR and SII were significantly elevated in patients with lower CrCl levels, while LCR and PNI were decreased. CAR showed a strong positive correlation with COVID-19 severity (r = 0.65, *p* < 0.001), and PNI was inversely correlated with mortality (r = −0.58, *p* < 0.001). Multivariate regression analysis indicated that lower CrCl levels, higher IS3 and CAR, and lower PNI were independent predictors of severe COVID-19 outcomes. Conclusions: CKD patients with lower CrCl levels have an amplified inflammatory response during COVID-19 infection, as evidenced by elevated inflammatory scores. The additional inflammatory scores, particularly CAR and PNI, may serve as valuable tools for risk stratification and management of COVID-19 in CKD patients. Early identification of patients with high CAR and low PNI could improve clinical outcomes through timely therapeutic interventions.

## 1. Introduction

The coronavirus disease 2019 (COVID-19) pandemic, caused by the severe acute respiratory syndrome coronavirus 2 (SARS-CoV-2), has significantly impacted global health, leading to substantial morbidity and mortality [1,2]. Patients with comorbidities such as chronic kidney disease (CKD) are particularly vulnerable to severe infection and adverse outcomes [3,4].

CKD affects approximately 10% of the global population and is associated with an increased risk of cardiovascular disease, infection, and mortality [5]. The interplay between CKD and COVID-19 poses a significant challenge as CKD patients often exhibit a heightened inflammatory state and impaired immune response [6,7]. These factors contribute to a greater susceptibility to infections and may exacerbate the severity of COVID-19 [8].

Inflammation plays a critical role in the pathogenesis of COVID-19, with severe cases often characterized by a “cytokine storm”, leading to acute respiratory distress syndrome (ARDS) and multi-organ failure [9,10]. CKD patients inherently have elevated levels of pro-inflammatory cytokines and immune dysregulation, which may predispose them to more severe disease manifestations [11,12].

Several inflammatory markers, such as C-reactive protein (CRP), interleukin-6 (IL-6), neutrophil-to-lymphocyte ratio (NLR), and ferritin, have been identified as predictors of COVID-19 severity [13,14]. Combining these laboratory parameters into inflammatory scores has shown promise in enhancing the predictive accuracy for disease outcomes [15]. Additionally, established indices like the systemic inflammatory index (SII), the C-reactive protein-to-albumin ratio (CAR), the lymphocyte-to-C-reactive protein ratio (LCR), and the prognostic nutritional index (PNI) have been utilized to assess inflammation and prognosis in various diseases, including COVID-19 [16,17,18]. However, the utility of such scores specifically in CKD patients with varying degrees of renal function remains underexplored.

Understanding the relationship between renal function, inflammatory response, and COVID-19 severity could inform clinical management and improve patient outcomes. Therefore, this study aimed to evaluate and compare the inflammatory status of COVID-19 patients with CKD, categorized by CrCl levels (<30, 30–60, and >60 mL/min). We calculated multiple inflammatory scores combining key laboratory parameters and assessed their correlation with disease severity and outcomes. The specific objectives were to determine if lower CrCl levels were associated with heightened inflammatory responses and to identify which inflammatory scores best predicted severe COVID-19 outcomes in this patient population.

## 2. Materials and Methods

### 2.1. Study Design and Population

This retrospective cohort study was conducted at the Victor Babes Hospital’s Department of Infectious Disease between April 2020 and April 2024. The study included 223 adult patients diagnosed with both COVID-19 and chronic kidney disease (CKD). Patients were stratified into three groups based on their creatinine clearance (CrCl) levels: CrCl < 30 mL/min (group 1, n = 41), CrCl 30–60 mL/min (group 2, n = 78), and CrCl > 60 mL/min (group 3, n = 104).

Inclusion criteria were patients aged 18 years or older, having confirmed SARS-CoV-2 infection via reverse transcription-polymerase chain reaction (RT-PCR) testing, having a history of diabetes mellitus or hypertension as an underlying cause of CKD, and having documented CKD with available CrCl measurements. Additionally, we excluded patients who received immunosuppressive medication or steroids. Exclusion criteria included patients on dialysis, those with acute kidney injury, or patients with incomplete medical records lacking essential laboratory data.

### 2.2. Data Collection and Laboratory Measurements

Data were gathered from electronic medical records, capturing demographic details such as age and sex, as well as information on comorbidities and clinical outcomes like hospitalization duration, ICU admission, the need for mechanical ventilation, and mortality rates. These variables were used to analyze the impact of patient characteristics on the disease severity and prognosis.

Upon hospital admission and 5 days into admission, a range of laboratory parameters were collected for each patient. These included C-reactive protein (CRP) measured via immunoturbidimetric assays, interleukin-6 (IL-6) quantified using enzyme-linked immunosorbent assay (ELISA), and the neutrophil-to-lymphocyte ratio (NLR) derived from complete blood count differentials. Ferritin levels were assessed with chemiluminescence immunoassay, while platelet count, absolute neutrophil count (ANC), and absolute lymphocyte count (ALC) were extracted from blood counts. Serum albumin was determined using a bromocresol green dye-binding method, and creatinine clearance (CrCl) was calculated using the Cockcroft–Gault equation adjusted for body surface area.

### 2.3. Inflammatory Scores

Several novel inflammatory scores were developed using the collected laboratory parameters to assess patient inflammation levels. These included inflammation score 1 (IS1), calculated as CRP multiplied by NLR, and inflammation score 2 (IS2), based on the product of IL-6 and ferritin levels. Inflammation score 3 (IS3) was derived from the combined values of CRP, IL-6, and NLR, while inflammation score 4 (IS4) averaged the values of CRP, IL-6, NLR, and ferritin.

Additionally, established inflammatory scores were computed for a more comprehensive analysis. The systemic inflammatory index (SII) was calculated as (platelet count × ANC)/ALC, while the C-reactive protein-to-albumin ratio (CAR) was determined by dividing CRP by serum albumin levels. The lymphocyte-to-C-reactive protein ratio (LCR) was obtained by dividing ALC by CRP, and the prognostic nutritional index (PNI) was assessed using the formula (10 × serum albumin) + (0.005 × ALC).

The systemic inflammatory index (SII) incorporated the platelet count, absolute neutrophil count (ANC), and absolute lymphocyte count (ALC) to assess overall inflammation. It was calculated using the following formula: (platelet count × ANC)/ALC. Higher SII values were associated with more severe disease, making it a comprehensive marker of inflammation and immune response. Another marker, the C-reactive protein-to-albumin ratio (CAR), used CRP and serum albumin levels, calculated as CRP divided by albumin. Elevated CAR levels were indicative of systemic inflammation and poor nutritional status, serving as a strong predictor of worse outcomes in COVID-19 patients.

The lymphocyte-to-C-reactive protein ratio (LCR), determined by dividing ALC by CRP, was used to evaluate the body’s inflammatory response; lower values suggested a more severe inflammatory state and poorer prognosis. Additionally, the prognostic nutritional index (PNI) assessed both nutritional and immune status. It was calculated as (10 × serum albumin) + (0.005 × ALC), with lower scores indicating a higher risk of severe disease and increased mortality. These indices collectively offered insights into the inflammatory and nutritional status of COVID-19 patients, helping predict clinical outcomes.

### 2.4. Statistical Analysis

A priori power analysis was performed to determine the necessary sample size for detecting differences in inflammatory scores among the three CrCl groups. Assuming a medium effect size (f = 0.25), a significance level of α = 0.05, and a desired power of 80%, the analysis indicated that at least 198 participants were required. Statistical analyses were performed using SPSS Statistics version 27.0 (IBM Corp., Armonk, NY, USA). Continuous variables were expressed as means ± standard deviation (SD) or medians with interquartile ranges (IQRs) as appropriate. Categorical variables were presented as frequencies and percentages. Comparisons among the three CrCl groups were conducted using one-way analysis of variance (ANOVA) for normally distributed continuous variables and Kruskal–Wallis tests for non-normally distributed variables. Chi-square tests were used for categorical variables. Pearson correlation coefficients were calculated to assess the relationships between inflammatory scores and clinical outcomes. Multivariate logistic regression analyses were performed to identify independent predictors of severe COVID-19 outcomes, adjusting for potential confounders such as age, sex, and comorbidities. A *p*-value of less than 0.05 was considered statistically significant.

## 3. Results

### Patient Demographics

A total of 223 patients were included in the study, with 41 patients in group 1 (CrCl < 30 mL/min), 78 patients in group 2 (CrCl 30–60 mL/min), and 104 patients in group 3 (CrCl > 60 mL/min). The mean age was significantly higher in group 1 (68.5 ± 12.3 years) compared with group 2 (65.2 ± 11.7 years) and group 3 (62.8 ± 10.9 years) (*p* = 0.02). Group 1 had a higher proportion of males (63.4%) compared with group 2 (56.4%) and group 3 (51.9%), but the difference was not statistically significant (*p* = 0.27). Comorbidities such as hypertension and diabetes mellitus were more prevalent in group 1 (*p* < 0.05). Table 1 summarizes the demographic and clinical characteristics of the study groups. Comorbid conditions such as hypertension and diabetes mellitus were more prevalent in group 1 (*p* = 0.01 and *p* = 0.004, respectively). ICU admission rates, the need for mechanical ventilation, and mortality were significantly higher in group 1 compared with the other group.

In the study, significant differences were observed across all measured laboratory parameters among the three creatinine clearance groups, as indicated by *p*-values less than 0.001. Patients in group 1 (CrCl < 30 mL/min) exhibited the highest levels of C-reactive protein at 85.2 ± 25.6 mg/L, interleukin-6 (IL-6) at 72.4 ± 18.5 pg/mL, and neutrophil-to-lymphocyte ratio (NLR) at 8.5 ± 2.7, which progressively decreased through group 2 (CrCl 30–60 mL/min) and group 3 (CrCl > 60 mL/min) to the lowest values in group 3, indicating a trend of reduced inflammatory status with improving renal function. Similarly, ferritin, D-dimer, creatinine, and absolute neutrophil count also followed this trend, while serum albumin and absolute lymphocyte count increased, suggesting better nutritional and immunological status in patients with higher CrCl (Table 2).

In the study, significant differences in inflammatory scores and related markers were observed across three groups categorized by creatinine clearance levels. The mean scores for inflammation score 1 (IS1) demonstrated a clear decreasing trend from group 1 (CrCl < 30, 724 ± 213) to group 3 (CrCl > 60, 326 ± 157), indicating lower inflammation levels in individuals with higher renal function. Similar patterns were noted for IS2, IS3, and IS4, where group 1 consistently exhibited the highest inflammatory scores (IS2: 39,862 ± 12,345; IS3: 52,126 ± 15,601; IS4: 179 ± 52) compared with the considerably lower scores in group 3 (IS2: 19,545 ± 8765; IS3: 16,278 ± 10,562; IS4: 101 ± 48).

Additional inflammatory and nutritional indices including the systemic inflammatory index (SII), C-reactive protein-to-albumin ratio, lymphocyte-to-C-reactive protein ratio, and prognostic nutritional index also varied significantly across the groups. For instance, the SII and CAR values were highest in group 1 (SII: 2.5 ± 0.7; CAR: 10.5 ± 3.2) and decreased progressively to group 3 (SII: 1.5 ± 0.5; CAR: 6.1 ± 2.4). In contrast, LCR and PNI showed an inverse relationship, with the lowest values in group 1 (LCR: 0.10 ± 0.03; PNI: 32.5 ± 4.6) and the highest in group 3 (LCR: 0.20 ± 0.05; PNI: 40.7 ± 5.9), as presented in Table 3.

Table 4 presents the mean changes in various inflammatory scores five days after admission for COVID-19 patients with chronic kidney disease, categorized into three groups based on CrCl levels. Across all inflammatory markers—IS1, IS2, IS3, IS4, SII, and CAR—group 1 demonstrated the most significant reductions (e.g., IS1 decreased by an average of −123 ± 20), followed by group 2 and then group 3, with all between-group comparisons yielding highly significant *p*-values (<0.001). Similarly, within each group, the changes from admission to day five were statistically significant (*p* < 0.001) for all scores. Additionally, the LCR and PNI scores, which reflect improvements in immune and nutritional status, showed positive mean differences across all groups (+0.016 ± 0.003 for group 1’s LCR and +4.61 ± 0.44 for PNI).

For inflammation scores IS1 through IS4, all demonstrated positive correlations with increased COVID-19 severity, higher rates of ICU admission, the need for mechanical ventilation, and mortality rates. Specifically, the correlation coefficients ranged from moderate to strong (r = 0.45 to r = 0.68), with IS3 showing the strongest correlations across all outcomes (COVID-19 severity r = 0.68 **, ICU admission r = 0.65 **, mechanical ventilation r = 0.63 **, mortality r = 0.62 **), indicating that higher IS3 values were significantly associated with worse clinical outcomes. Positive correlations were noted for SII and CAR with all clinical outcomes, suggesting that higher levels of systemic inflammation were predictive of poorer patient prognosis. Conversely, LCR and PNI showed negative correlations (e.g., COVID-19 severity: LCR r = −0.58 **, PNI r = −0.60 **), indicating that higher values in these indices, reflecting better immune response and nutritional status, were associated with less severe disease and lower rates of adverse outcomes (Table 5).

Creatinine clearance less than 30 mL/min was significantly associated with a higher risk of negative clinical outcomes, showing an odds ratio (OR) of 3.2 with a 95% confidence interval (CI) from 2.0 to 5.2 and a *p*-value less than 0.001. This finding indicated that severely reduced renal function significantly increased the risk of poor outcomes. Similarly, each unit increase in the C-reactive protein-to-albumin ratio (CAR) resulted in a 25% increase in the odds of adverse outcomes (OR 1.25, 95% CI 1.15–1.36, *p* < 0.001). Furthermore, each unit decrease in the prognostic nutritional index (PNI) was associated with an 18% increase in adverse outcomes (OR 1.18, 95% CI 1.10–1.27, *p* < 0.001), emphasizing the role of nutritional status in patient prognosis. The analysis also identified diabetes mellitus as a significant factor, with an odds ratio of 1.6 (95% CI 1.1–2.6, *p* = 0.005), indicating a higher risk of negative outcomes among diabetic patients. However, age showed a minimal effect on the likelihood of adverse outcomes per year increase (OR 1.02, 95% CI 1.00–1.04, *p* = 0.33), as described in Table 6.

## 4. Discussion

### 4.1. Analysis of Findings

This study evaluated the inflammatory status of COVID-19 patients with CKD, stratified by CrCl levels, and assessed the predictive value of multiple inflammatory scores. The findings indicated that patients with lower CrCl levels (<30 mL/min) exhibited a heightened inflammatory response and poorer nutritional status, as evidenced by elevated levels of CRP, IL-6, NLR, ferritin, SII, and CAR and decreased LCR and PNI. IS3 and CAR emerged as strong positive predictors of severe COVID-19 outcomes, while PNI was inversely associated with disease severity and mortality.

Our results align with previous studies that have identified CKD as a risk factor for severe COVID-19 outcomes [4,19,20]. The elevated inflammatory markers observed in patients with lower renal function corroborate findings that CKD is associated with a chronic pro-inflammatory state [21]. The significant correlations of CAR and PNI with clinical outcomes are consistent with other research demonstrating the prognostic value of these indices in COVID-19 and other inflammatory conditions [22,23].

The strong predictive value of CAR suggests that combining CRP with serum albumin enhances the assessment of inflammatory status and nutritional condition, which are critical factors in the prognosis of COVID-19 patients [24]. Similarly, the inverse relationship between PNI and disease severity underscores the importance of nutritional status in immune competence and recovery [25].

In a similar manner, the study by Russo et al. [26] found that elevated creatinine levels at hospital admission were significantly associated with increased mortality in COVID-19 patients, identifying a critical cutoff value of 1.12 mg/dL for predicting mortality (OR 2.233, 95% CI: 1.373–3.634, *p* < 0.001). This observation underscores the prognostic value of renal function at the onset of hospitalization. Comparatively, the prospective cohort study conducted by Al Rumaihi et al. [27] explored the renal impacts of mild and asymptomatic COVID-19 in healthy young adults, demonstrating that mild pneumonia due to COVID-19 led to significant, although mild, renal involvement indicated by elevated serum creatinine levels at diagnosis and persisting up to 6 months post-infection (beta  =  12.836 to 14.345, *p*-values ranging from 0.019 to 0.040). Both studies highlight the crucial role of kidney function as a determinant of COVID-19 severity and progression, albeit in distinctly different patient populations—Russo et al. in a broader, more diverse group likely at higher risk and Al Rumaihi et al. [27] in a narrowly defined, healthier cohort. Thus, these findings collectively suggest that monitoring renal parameters could be essential for assessing COVID-19 impact and outcomes across varied demographics and disease severities.

In their study, Alfano et al. [28] highlighted the prognostic significance of even slight changes in serum creatinine during the early stage of hospitalization in COVID-19 patients, finding that changes within a range of −0.05 to +0.05 mg/dL were associated with differing survival rates, suggesting that modest renal dysfunction upon admission could predict poorer outcomes. Similarly, the study by Komaru and Doi [29] also underscored the importance of renal changes in COVID-19 patients, noting that approximately 10% of hospitalized patients developed acute kidney injury (AKI), which was significantly linked to adverse outcomes. Both studies emphasize the critical need for the early recognition and monitoring of kidney function in COVID-19 patients, although Alfano et al. specifically demonstrated that even minimal increases in serum creatinine (more than +0.05 mg/dL) during the initial 24 h significantly correlated with higher in-hospital mortality and AKI rates, challenging the conventional AKI diagnostic threshold of a 0.3 mg/dL increase. This finding suggests a potential need for revising AKI diagnostic criteria in the context of COVID-19, to include smaller changes in serum creatinine, thereby facilitating earlier intervention and potentially improving patient outcomes.

In their cross-sectional study, Žulpaitė et al. [30] investigated the correlation between COVID-19 infection and kidney damage in a regional university hospital, finding that patients who developed acute kidney injury or whose chronic kidney disease was complicated by AKI had significantly higher mortality rates and longer hospital stays. Specifically, the mortality rate among patients with AKI was 7.79 times higher than among those without these conditions (*p* < 0.001). In a similar manner, the prospective pilot study by Temiz et al. [31] assessed kidney damage markers in hospitalized COVID-19 patients and identified a strong predictive value for survival outcomes associated with these markers. Particularly, they highlighted the urine Kidney Injury Molecule-1 (KIM-1)/creatinine ratio as a significant predictor of COVID-19 specific death in their multivariable analysis (OR 6.11; 95% CI: 1.22–30.53; *p* = 0.02), reinforcing the prognostic importance of kidney injury markers in assessing the severity and potential mortality of COVID-19 patients. Both studies underscore the severe implications of renal complications in COVID-19, suggesting that kidney function markers are crucial in predicting patient outcomes and necessitate careful monitoring during the course of the disease.

Overall, it is noted that the identification of CAR and PNI as significant predictors of severe outcomes has practical implications. Clinicians can utilize these scores to stratify risk among CKD patients with COVID-19, enabling targeted interventions and closer monitoring. Early identification of high-risk patients may facilitate timely therapeutic strategies, such as aggressive anti-inflammatory treatments and nutritional support, potentially improving prognosis and reducing mortality.

The role of underlying health conditions, particularly diabetes, in exacerbating the severity of COVID-19 has been well documented in the literature. In our study, we observed a notable increase in inflammatory markers among CKD patients with diabetes, which correlated with more severe COVID-19 outcomes. This finding is consistent with previous research that suggests diabetes can exacerbate the inflammatory response during COVID-19, potentially due to an already compromised immune system that struggles to manage viral infections effectively. For instance, a study by Gupta et al. [32] found that diabetes was associated with an increased risk of severe COVID-19 and poor outcomes, largely due to enhanced inflammatory activity. Similarly, research by Maddaloni et al. [33] highlighted that patients with diabetes and COVID-19 exhibited significantly higher levels of inflammatory markers such as CRP and IL-6, which were predictive of ICU admission and mortality. Our study further substantiates these findings by demonstrating a clear link between inflammatory markers and disease severity in a CKD population predominantly affected by diabetes and hypertension, suggesting that management strategies in such patients should address not only viral infection but also the underlying metabolic derangements contributing to inflammation.

Nevertheless, the interplay between chronic kidney disease and COVID-19 severity is underpinned by several biological mechanisms that exacerbate patient outcomes. CKD is characterized by a state of chronic inflammation and immune dysregulation, which can amplify the hyperinflammatory response observed in severe COVID-19 cases. Patients with reduced renal function often exhibit elevated levels of pro-inflammatory cytokines, such as IL-6 and CRP, which are further intensified by SARS-CoV-2 infection, leading to a more pronounced cytokine storm and increased disease severity [34]. Additionally, impaired kidney function may result in altered expression of angiotensin-converting enzyme 2, the primary receptor for viral entry, potentially facilitating greater viral load and tissue damage [35]. The accumulation of uremic toxins in CKD also compromises immune cell function, diminishing the body’s ability to effectively respond to viral infections. Moreover, the high prevalence of comorbid conditions like hypertension and diabetes in CKD patients further contributes to the heightened risk of adverse COVID-19 outcomes. Understanding these underlying mechanisms highlights the need for targeted clinical management strategies to mitigate the compounded effects of CKD and COVID-19, ultimately improving patient prognosis.

### 4.2. Study Limitations

This study has several limitations. First, its retrospective design may introduce selection bias, and the findings were based on data from a single center, which may limit generalizability. Second, we excluded patients on dialysis and those with acute kidney injury, which may have provided additional insights into the inflammatory status of patients with varying renal replacement therapies. Third, the study did not account for the effects of specific COVID-19 treatments or CKD management strategies, which could have influenced the outcomes.

## 5. Conclusions

In conclusion, CKD patients with lower CrCl levels exhibit a heightened inflammatory response and poorer nutritional status during COVID-19 infection, leading to worse clinical outcomes. The inflammatory scores, particularly CAR and PNI, serve as strong predictors of disease severity and mortality. These findings highlight the importance of assessing both inflammatory and nutritional status in CKD patients with COVID-19 and suggest that CAR and PNI could be valuable tools for risk stratification. Early identification and targeted management of high-risk patients may improve clinical outcomes. Future studies should validate these findings in larger, multicenter cohorts and explore the utility of these inflammatory scores in guiding therapeutic decisions.

## Figures and Tables

**Table 1:** Demographic and clinical characteristics of the study groups.

Variables	Group 1 (CrCl < 30) (n = 41)	Group 2 (CrCl 30–60) (n = 78)	Group 3 (CrCl > 60) (n = 104)	*p*-Value
Age (years)	68.5 ± 12.3	65.2 ± 11.7	62.8 ± 10.9	0.02
Male sex (%)	63.4%	56.4%	51.9%	0.27
Hypertension (%)	85.4%	76.9%	65.4%	0.01
Duration of hypertension, median (IQR)				
Diabetes mellitus (%)	68.3%	53.8%	42.3%	0.004
Duration of diabetes, median (IQR)				
BMI (kg/m^2^)	28.7 ± 4.5	27.9 ± 4.2	26.5 ± 3.9	0.03
ICU admission (%)	39.0%	28.2%	15.4%	<0.001
Mechanical ventilation (%)	26.8%	17.9%	8.7%	<0.001
Mortality (%)	31.7%	21.8%	9.6%	<0.001

BMI—body mass index; ICU—intensive care unit; IQR—interquartile range.

**Table 2:** Laboratory parameters among the CrCl groups.

Parameters	Group 1 (CrCl < 30) (n = 41)	Group 2 (CrCl 30–60) (n = 78)	Group 3 (CrCl > 60) (n = 104)	*p*-Value
CRP (mg/L)	85.2 ± 25.6	72.5 ± 20.3	60.3 ± 18.7	<0.001
IL-6 (pg/mL)	72.4 ± 18.5	60.8 ± 16.2	50.1 ± 14.7	<0.001
NLR	8.5 ± 2.7	6.9 ± 2.1	5.4 ± 1.8	<0.001
Ferritin (μg/L)	550.2 ± 150.6	470.3 ± 130.4	390.5 ± 120.7	<0.001
D-dimer (μg/mL)	1.5 ± 0.5	1.2 ± 0.4	0.9 ± 0.3	<0.001
Creatinine (mg/dL)	3.2 ± 0.8	2.1 ± 0.6	1.2 ± 0.4	<0.001
Serum albumin (g/L)	31.3 ± 4.6	34.0 ± 5.3	38.6 ± 5.5	<0.001
Platelet count (×10⁹/L)	216 ± 62	238 ± 51	257 ± 53	<0.001
ANC (×10⁹/L)	8.0 ± 2.2	6.4 ± 2.0	5.3 ± 1.7	<0.001
ALC (×10⁹/L)	0.8 ± 0.3	1.1 ± 0.4	1.2 ± 0.4	<0.001

CRP—C-reactive protein; IL-6—interleukin-6; NLR—neutrophil-to-lymphocyte ratio; ANC—absolute neutrophil count; ALC—absolute lymphocyte count.

**Table 3:** Comparison of inflammatory scores among CrCl groups.

Inflammatory Scores (at Admission)	Group 1 (CrCl < 30) (n = 41)	Group 2 (CrCl 30–60) (n = 78)	Group 3 (CrCl > 60) (n = 104)	*p*-Value
IS1	724 ± 213	502 ± 188	326 ± 157	<0.001
IS2	39,862 ± 12,345	28,594 ± 10,876	19,545 ± 8765	<0.001
IS3	52,126 ± 15,601	30,149 ± 12,082	16,278 ± 10,562	<0.001
IS4	179 ± 52	140 ± 45	101 ± 48	<0.001
SII (×10⁹/L)	2.5 ± 0.7	2.0 ± 0.6	1.5 ± 0.5	<0.001
CAR	10.5 ± 3.2	8.2 ± 2.8	6.1 ± 2.4	<0.001
LCR (×10⁻^3^)	0.10 ± 0.03	0.15 ± 0.04	0.20 ± 0.05	<0.001
PNI	32.5 ± 4.6	36.3 ± 5.0	40.7 ± 5.9	<0.001

IS1—inflammation score 1; IS2—inflammation score 2; IS3—inflammation score 3; IS4—inflammation score 4; SII—systemic inflammatory index; CAR—C-reactive protein-to-albumin ratio; LCR—lymphocyte-to-C-reactive protein ratio; PNI—prognostic nutritional index.

**Table 4:** Mean differences in inflammatory scores at 5 days from admission among CrCl groups.

Inflammatory Scores (5 Days Since Admission)	Group 1 (CrCl < 30) (n = 41)	Group 2 (CrCl 30–60) (n = 78)	Group 3 (CrCl > 60) (n = 104)	*p*-Value (Between Groups)	*p*-Value (Within Groups)
IS1	−123 ± 20	−92 ± 18	−47 ± 15	<0.001	<0.001 (All Groups)
IS2	−5433 ± 1201	−4023 ± 1034	−2947 ± 812	<0.001	<0.001 (All Groups)
IS3	−7823 ± 1605	−6021 ± 1234	−2463 ± 1059	<0.001	<0.001 (All Groups)
IS4	−29 ± 5	−21 ± 4	−14 ± 4	<0.001	<0.001 (All Groups)
SII (×10⁹/L)	−0.42 ± 0.07	−0.31 ± 0.06	−0.24 ± 0.05	<0.001	<0.001 (All Groups)
CAR	−2.13 ± 0.34	−1.63 ± 0.28	−0.93 ± 0.24	<0.001	<0.001 (All Groups)
LCR (×10⁻^3^)	+0.016 ± 0.003	+0.021 ± 0.004	+0.027 ± 0.005	<0.001	<0.001 (All Groups)
PNI	+4.61 ± 0.44	+4.77 ± 0.49	+4.93 ± 0.58	<0.001	<0.001 (All Groups)

IS1—inflammation score 1; IS2—inflammation score 2; IS3—inflammation score 3; IS4—inflammation score 4; SII—systemic inflammatory index; CAR—C-reactive protein-to-albumin ratio; LCR—lymphocyte-to-C-reactive protein ratio; PNI—prognostic nutritional index.

**Table 5:** Correlations between inflammatory scores and clinical outcomes.

Clinical Outcomes	IS1	IS2	IS3	IS4
COVID-19 severity	r = 0.54 **	r = 0.58 **	r = 0.68 **	r = 0.50 *
ICU admission	r = 0.50 *	r = 0.53 **	r = 0.65 **	r = 0.47 *
Mechanical ventilation	r = 0.48 *	r = 0.51 **	r = 0.63 **	r = 0.45 *
mortality	r = 0.49 *	r = 0.55 **	r = 0.62 **	r = 0.46 *
**Clinical Outcomes**	**SII**	**CAR**	**LCR**	**PNI**
COVID-19 severity	r = 0.62 **	r = 0.65 **	r = −0.58 **	r = −0.60 **
ICU admission	r = 0.60 **	r = 0.63 **	r = −0.55 *	r = −0.57 **
Mechanical ventilation	r = 0.58 **	r = 0.61 **	r = −0.53 *	r = −0.55 *
Mortality	r = 0.59 *	r = 0.64 **	r = −0.56 *	r = −0.58 **

ICU—intensive care unit; SII—systemic inflammatory index; CAR—C-reactive protein-to-albumin ratio; LCR—lymphocyte-to-C-reactive protein ratio; PNI—prognostic nutritional index; *—*p*-value < 0.05; **—*p*-value < 0.001.

**Table 6:** Multivariate regression analysis.

Variables	Odds Ratio	95% CI	*p*-Value
CrCl < 30 mL/min	3.2	2.0–5.2	<0.001
IS3 (per unit increase)	1.01	1.008–1.012	0.048
CAR (per unit increase)	1.25	1.15–1.36	<0.001
PNI (per unit decrease)	1.18	1.10–1.27	<0.001
Age (per year increase)	1.02	1.00–1.04	0.33
Diabetes mellitus	1.6	1.1–2.6	0.005

CrCl—creatinine clearance; IS3—inflammation score 3; CAR—C-reactive protein-to-albumin ratio; PNI—prognostic nutritional index; CI—confidence interval.

## Data Availability

The data presented in this study are available on request from the corresponding author due to legal and ethical reasons.
